# Painong San, a Traditional Chinese Compound Herbal Medicine, Restores Colon Barrier Function on DSS-Induced Colitis in Mice

**DOI:** 10.1155/2021/2810915

**Published:** 2021-12-20

**Authors:** Xuelin Rui, Jiacheng Li, Ye Yang, Li Xu, Yang Liu, Mengmeng Zhang, Dengke Yin

**Affiliations:** ^1^School of Pharmacy, Anhui University of Chinese Medicine, Hefei 230012, China; ^2^Anhui Provincial Key Laboratory of Research & Development of Chinese Medicine, Hefei 230012, China; ^3^Key Laboratory of Xin'an Medicine, Ministry of Education, Hefei 230012, China; ^4^Institute of Pharmaceutics, Anhui Academy of Chinese Medicine, Hefei 230012, China

## Abstract

**Objective:**

The intestinal barrier decreases in colitis and restores the integrity of the mucosal barriers that could be used for the treatment of colitis. Painong San (PNS), a traditional Chinese compound herbal medicine originally recorded in “Jingui Yaolve” by Zhongjing Zhang in the Later Han Dynasty, is often used in China and Japan to treat various purulent diseases including intestinal carbuncle. This study was to investigate the effect of PNS on mucosal barrier function in mice with DSS-induced colitis and its related mechanisms.

**Methods:**

BALB/C mice were given 3% DSS to induce colitis. The body weight and stool status of the mice were recorded daily, and the histopathological changes of the colon were observed after execution. The permeability of the intestinal mucosa was measured by fluorescein isothiocyanate-dextran 4000, the change of intestinal microbiota was measured by 16S rDNA, and the tight junction-related proteins and Muc-2 were investigated by immunohistochemical or immunofluorescence. The possible signaling pathways were detected by western blot.

**Results:**

Compared with the control group, the composition of the microbiota in the PNS group was close to that of the normal group, the number of goblet cells was improved, and the mucosal permeability was significantly reduced. PNS could upregulate the expression of tight junction-related proteins (ZO-1, claudin-1, and occludin) and Muc-2, and at the same time, regulate the Notch pathway.

**Conclusion:**

PNS could effectively improve the mucosal barrier function through multiple ways, including restoring the balance of intestine flora, enhancement of the mucous layer barrier, and mechanical barrier function. These protective effects may relate to inhibiting the Notch signaling pathway activated by DSS.

## 1. Introduction

Colitis, usually with intestinal mucosal congestion, edema, bleeding, and other symptoms [1–3], is a disease that seriously threatens the health of modern people. Although the underlying pathogenic factors of colitis are not yet clear, more and more studies have shown that a healthy intestine is closely related to a good mucosal barrier. This provides a new idea for the treatment of colitis.

The intestinal epithelial barrier (intestinal epithelial cells and their tight junctions), the mucus barrier (intestinal mucosal goblet cells, mucin) secreted by epithelial cells, and the biological barrier (intestinal microbiota) are important components of the intestinal mucosal barrier [4, 5]. Although they are relatively independent in composition, they have complicated internal connections, forming a huge three-dimensional defense system. In the early stages of colitis, the balance of symbiotic microbiota changes, leading to some nonrepresentative flora occupying a dominant position [6, 7]. Some of the bacterial secretions promote the malfunction of TJs [8], and the permeability and integrity of the intestinal tract change, allowing the bacterial flora and its products to penetrate the mucosa. Bacteria contact with it, resulting in incomplete cell membranes, destroying the epithelial barrier, affecting the production of mucin, and loss of GC. Eventually, these led to further adverse reactions. Therefore, it is of great significance for colitis to find a multichannel method to restore the intestinal mucosal barrier function.

PNS, originally recorded in “Jingui Yaolve” by Zhongjing Zhang in the Later Han Dynasty, is a formula consisting of Aurantii Fructus Immaturus, Radix Paeoniae Alba, and Radix Platycodi. PNS (named Hainosan in Japanese) is also the OTC medicinal product in Kampo in Japanese to treat various purulent diseases including intestinal carbuncle [9]. Combining modern research, it is found that its formula has good effects in anti-inflammatory [10, 11], antibacterial[12–14], and regulating intestinal secretion [15]. But there is no scientific report to show its curative effect on intestinal inflammatory diseases. In this study, a mouse model of colitis was established to study the protective effect of PNS on ulcerative colitis from the intestinal mucosal barrier and its regulatory effect on the Notch signaling pathway.

## 2. Materials and Methods

### 2.1. Animals

Male BALB/C mice, weighing 20 ± 5 g, were provided by the Animal Experiment Center of Anhui Medical University. All mice were kept in a specific-pathogen-free (SPF) animal house with free access to food and water and a 12 h light/dark cycle. The study followed the “Guide for the Care and Use of Laboratory Animals” and was approved by the Experimental Animal Ethics Committee of Anhui University of Traditional Chinese Medicine (AHUCM-mouse-2021028).

### 2.2. Materials, Drugs, and Reagents

PNS is composed of *Aurantii Fructus Immaturus* (dried young fruit of *Citrus aurantium* L. or *Citrus sinensis Osbeck*), *Radix Paeoniae Alba* (tuberous root of *Paeonia lactiflora Pall.*), and *Platycodonis Radix* (root of *Platycodonis Radix*) at a ratio of 5 : 5: 2. *Aurantii Fructus Immaturus* (200601), *Radix Paeoniae Alba* (200320), and *Platycodonis Radix* (200312) were purchased from Anhui Guanghe Medicine Co. Ltd. (Bozhou, China). All of them were certified by Professor Shoujin Liu of Anhui University of Chinese Medicine. The three raw materials of PNS were powdered and sieved and then mixed with water according to the ratio for intragastric administration. DSS was purchased from Meilun Biotechnology Co. (LLC, France); hesperidin (DST190716-038), neohesperidin (DST190929-039), naringin reference substance (DST191011-099), and paeoniflorin (DST190303-070) reference substance (mass fraction > 98%) were purchased from Le Meitian Pharmaceutical Co. Ltd. (Chengdu, China); sulfasalazine (09190515) was purchased from Xinyi Tianping Pharmaceutical Co. (Shanghai, China); ZO-1 antibody (bs-1329R) was purchased from Bioss Biotechnology Co. (Beijing, China); and occludin antibody (502601), claudin-1 antibody (343203), Notch antibody (380355), Hes1 antibody (381205), and Math1 antibody (382388) were all purchased from ZEN-Biotechnology Co. (Beijing, China). FITC-Dextran 4 (4 kDa) (60842-46-8) was purchased from MK-Biotechnology Co. (Shanghai, China).

### 2.3. Animal Modeling and Grouping

According to the method described in the literature [16], an acute colitis model was induced in BALB/C mice. After one week of adaptive feeding, 72 mice were randomly divided into the following 6 groups (*n* = 12 groups in each group): the normal group, the model group (DSS), the sulfasalazine group (DSS + SASP), the PNS low-dose (DSS + PNS-L) group, the PNS medium-dose (DSS + PNS-M) group, and the PNS high-dose (DSS + PNS-H) group. Mice could get free feed and drinking water for 1 week. From the first day, the DSS + SASP group received oral SASP (200 mg/kg), and the groups of DSS + PNS-L, DSS + PNS-M, and DSS + PNS-H received doses of 0.8 g/kg, 1.6 g/kg, and 3.2 g/kg of PNS, respectively. A suspension is prepared by dispersing the powders in distilled water. The normal group and the model group each received the same dose of distilled water. All mice were administered once a day at a fixed time.

### 2.4. PNS Chemical Composition Analysis

#### 2.4.1. Sample Preparation for UPLC Analysis

*Aurantii Fructus Immaturus*, *Radix Paeoniae Alba*, and *Platycodonis Radix* were powdered, respectively, then passed through a 100-mesh sieve, accurately weighed, and mixed according to the ratio of the composition. 70% methanol is added to the mix, sonicated for 30 min, centrifuged at 5400 rpm/min for 10 min, and filtered to obtain the additional filtrate (the crude drug content is 0.16 g/ml), and the filtrate is diluted 20 times with a solvent and then enters the UPLC system for testing.

#### 2.4.2. UPLC Analysis

Separated using the Thermo Scientific Syncronis C_18_ (100 mm × 2.1 mm, 1.7 *μ*m) chromatographic column. Column temperature 35°C, injection volume 2 *μ*L, mobile phase methanol (A) and water (B), gradient elution: 0∼3 min 80%B; 3∼4 min 65%B; 4∼9 min 65%B; 9∼13 min 50%B; 13∼14 min 25%B; 14∼17.5 min 25%B; 17.5∼17.6 min 80%B; and 17.6∼22 min 80%B.

### 2.5. Intestinal Permeability

The intestinal permeability was measured as described in [17]. The mice were fasted for 4 hours and given FITC-dextran 4 kDa (200 mg/kg body weight). 4 hours later, blood was taken from the inner canthus, and the blood was placed in the dark at 4°C at 2000 r/min and centrifuged for 10 minutes to collect serum. Serum and PBS buffer (pH = 7.4) were diluted at a ratio of 1 : 3; the concentration of FITC-D (*λ*exc:495 nm and *λ*em:520 nm) was detected using a fluorescence spectrophotometer.

### 2.6. Histopathological Analysis

Colon sections were fixed in 10% buffered formalin and embedded in paraffin. Colon sections (4 *μ*m) were then deparaffinized with xylene and rehydrated with hematoxylin and eosin (H&E). Inflammation and crypt damage were assessed using a light microscope and the grading of histological damage was evaluated as previously described.

### 2.7. Immunohistochemical (IHC) or Immunofluorescence (IF) Analyses

The immunohistochemical staining and immunofluorescence staining steps are basically the same, but some steps are modified. In brief, paraffin-embedded slides were deparaffinized and exposed to antigen by microwave heating in citrate buffer for 20 minutes. After washing with PBS, the slides were incubated with 3% H_2_O_2_ and goat serum at room temperature for 10 min and 15 min, respectively. Add rabbit anti-claudin-1 (1 : 200) and Muc-2 (1 : 200) and incubate overnight at 4°C. Add goat anti-rabbit secondary antibody and incubate for 30 min at room temperature, followed by DAB color development and hematoxylin staining. Immunofluorescence staining uses occludin-1 (1 : 200), ZO-1 (1 : 200), and IgG-Cy3 (1 : 300) as primary antibodies and fluorescently labeled secondary antibodies. The image was taken with a fluorescent inverted microscope.

### 2.8. 16S rDNA Sequence Analysis

The intestinal contents were collected and frozen at −80°C. The microbial DNA was extracted using the E. Z.N.A.® soil DNA Kit (Omega Bio-Tek, USA) according to the manufacturer's protocols, qualified by 1% agarose gel electrophoresis, and quantified by the UV-Vis spectrophotometer (NanoDrop 2000, Thermo Fisher Scientific, USA). The V3–V4 hypervariable regions of the bacteria 16S rRNA gene were amplified with primers 338 F (5′ACTCCTACGGGAGGCAGCAG-3′) and 806 R (5′-GGACTACHVGGGTWTCTAAT-3′) by a polymerase chain reaction (PCR) system (ABI GeneAmp® 9700, ABI, USA). Amplicons were then purified by gel extraction (Axygen Biosciences, Axygen, USA), quantified using QuantiFluor-ST (Promega, USA), pooled in equimolar concentrations, and paired-end sequenced using an Illumina MiSeq instrument (Illumina, USA). Fastp (V0.19.6) was used for original sequence quality control, and Flash (V1.2.11) was used for stitching. The bases with read tail quality value lower than 20 and those under 50 bp were filtered. Paired reads were spliced (merged) into a sequence, and the maximum error ratio allowed in the overlap area of the stitching sequence was 0.2. UPARSE (V7.0.1090) was used to cluster the operational taxonomic unit (OTU) with 97% homology. All optimized sequences were mapped to the representative OTU sequences. The sequences with 97% homology to the representative OTU sequences were selected to generate the OTU table. The species of each sequence were classified by the RDP classifier (V2.11) and compared with the Silva database (V132). The alpha diversity, including Sobs and Shannon index, was calculated by the mothur (V1.30.1). The unweighted pair-group method with arithmetic mean (UPGMA) algorithm (PCoA) was used to build the tree structure for the principal co-ordinates analysis. Statistical Product and Service Solutions (SPSS, V25.0) was used to treat the data in the PCoA score map with multivariate analysis of variance.

### 2.9. Western Blot Analysis

The tissue was extracted using RIPA lysis buffer with the protease inhibitor phenylmethanesulfonyl fluoride. The BCA protein assay kit was used to measure the protein concentration according to the manufacturer's instructions. An equal amount of protein was separated on an SDS-PAGE gel, and then transferred to a 0.45 *μ*m PVDF membrane according to the standard protocol. At room temperature, the membrane was blocked in 5% milk in TBST buffer for 1 hour and then incubated with the primary antibody (the primary antibodies and their dilution concentration were as follows: Notch (1 : 3000), HES1(1 : 2000), and Math (1 : 2000)) at 4°C overnight. After incubating with the secondary antibody for 1 hour at room temperature, the ECL reagent was used to detect the protein. Three independent repetitions were performed.

### 2.10. Statistical Analyses

Values are expressed as mean ± standard error (SEM). When appropriate, a two-tailed paired *t*-test was used to distinguish significant differences between groups. *p* < 0.05 was considered to indicate statistically significant differences.

## 3. Results

### 3.1. UPLC Analysis

**Figure 1:**
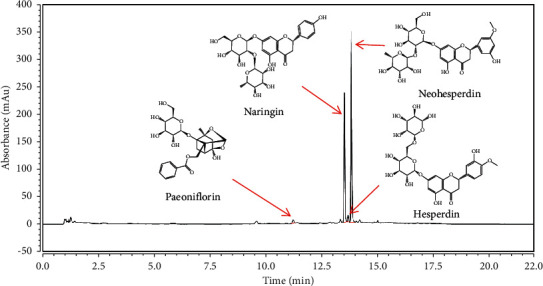
The UPLC chromatogram of PNS.

Four main components were separated and identified from PNS by UPLC with standard substance (Figure 1), which are paeoniflorin, naringin, hesperidin, and neohesperidin. Quantitative analysis showed that the contents of paeoniflorin, naringin, hesperidin, and neohesperidin were 0.52 ± 0.06 mg/g, 1.31 ± 0.07 mg/g, 0.22 ± 0.04 mg/g, and 1.36 ± 0.12 mg/g, respectively [18].

### 3.2. PNS Promotes Recovery of DSS‐Induced Colitis in Mice

**Figure 2:**
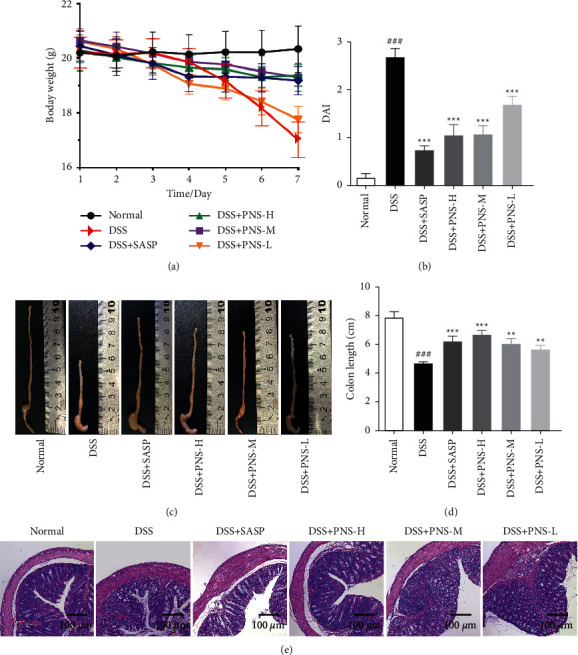
PNS promotes recovery of DSS‐induced colitis in mice. (a) Daily changes in body weight of each group during the course of the disease; (b) evaluation of the DAI scores of each group after execution; (c) observation of the colon; (d) measurement of the length of the colon in each group; (e) H&E staining of tissues, *n* = 12. ^#^*p* < 0.05, ^##^*p* < 0.01, and ^###^*p* < 0.001, versus the control group, ^*∗*^*p* < 0.05, ^*∗∗*^*p* < 0.01, and ^*∗∗∗*^*p* < 0.001 versus the DSS group.

In order to evaluate the therapeutic effect of PNS on colonic epithelial injury and colitis caused by DSS, we used 3% DSS to induce mice to establish a colitis model for 7 days to further determine whether PNS could improve colitis. Compared with the normal group, the DSS group showed continuous weight loss, accompanied by severe diarrhea and fecal occult blood. On the contrary, the weight of the mice given SASP and PNS recovered significantly, and the DAI score of the mice was significantly improved (Figures 2(a) and 2(b)). In addition, the administration of DSS can cause varying degrees of colon shortening. Compared with the DSS group, mice in the SASP and PNS groups had significantly longer colon lengths (Figures 2(c) and 2(d)). HE results (Figure 2(e)) showed that the colon in the normal group had a clear texture, complete morphology, no hyperemia, and a neat arrangement of intestinal epithelial cells in the mucosal layer. The colon tissue of mice in the model group was obviously damaged, and the pathological changes were obvious. The main manifestations were disordered texture, porous structure, irregular arrangement of intestinal epithelial cells, and fuzzy goblet cells. On the contrary, the crypt structure of the mice treated with SASP, H, and M groups was relatively complete, with less epithelial deformation and less inflammatory cell infiltration. The L group showed obvious tissue damage, intestinal mucosal damage, and death and shedding of intestinal epithelial cells. This indicates that PNS has a certain therapeutic effect on colitis with intestinal epithelial cell damage.

### 3.3. PNS Regulates the Microbial Barrier of Mice with Colitis

**Figure 3:**
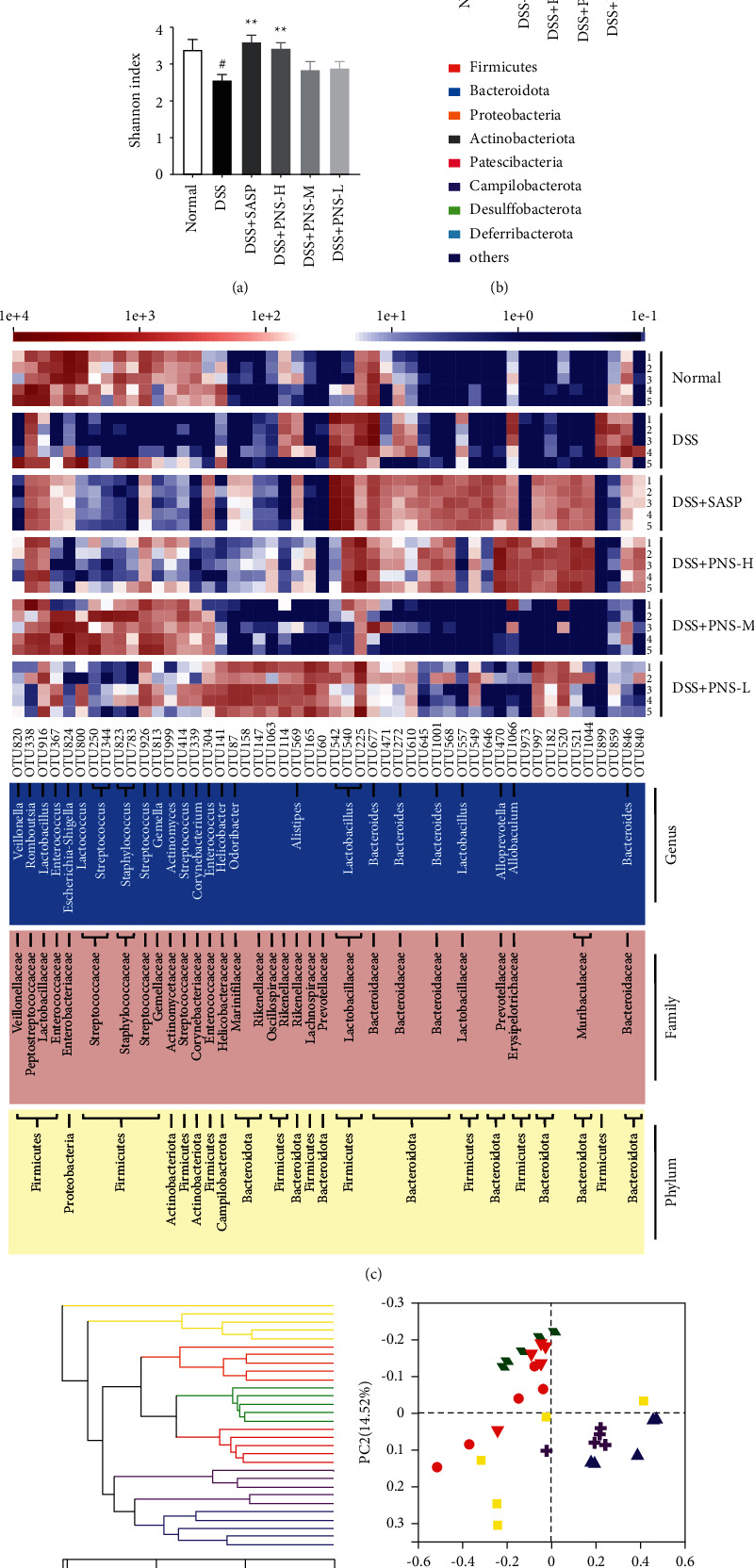
PNS affects the composition of the microbiota in mice with colitis. (a) Diversity index diagram of each group based on OTU level; (b) phylum level analysis of intestinal bacteria in different groups of mice; (c) PNS treatment of DSS induction colitis causes 50 OTUs changes in the heatmap. The phylum, family, and genus names of OTUs are displayed on the right panel; (d) sample similarity tree of each group; (e) primary coordinate analysis (PCoA) of each group of samples , using weight-unifrac distance algorithm; *n* = 5. ^#^*p* < 0.05, ^##^*p* < 0.01, and ^###^*p* < 0.001, versus the normal group, ^*∗*^*p* < 0.05, ^*∗∗*^*p* < 0.01, and ^*∗∗∗*^*p* < 0.001 versus the DSS group.

As we all know, the intestinal mucosal barrier separates the intestinal microbiota from the host immune system and maintains the normal balance in the intestine. However, the intestinal microbiota, as a part of the mucosal barrier, directly or indirectly participates in the regulation of the mucosal barrier and has long been in a mutually beneficial and win-win relationship with the host [19]. Therefore, adjusting the composition and proportion of microorganisms is a necessary condition for protecting the mucosal barrier. Compared with the normal group, the overall number of OTUs observed in the DSS group decreased, which was reversed by PNS treatment (Figure 3(a)). The three main bacteria at the level of intestinal flora are Firmicutes, Bacteroides, and Proteobacteria. Compared with the control group, during the development of colitis, the abundance of Bacteroides phylum decreased, while the abundance of Firmicutes and Proteobacteria increased (Figure 3(b)). During the DSS induction process, PNS significantly changed the OTUs. We analyzed the first 50 OTUs with significant changes. It is worth noting that the genus-level analysis shows that 4 OTUs are from the genus Bacteroides. In addition, PNS can also reduce the types of opportunistic pathogens, such as *Streptococcus* (4 OTUs) while increase the abundance of beneficial bacteria, such as lactic acid bacteria (5 OTUs) and *Alistipes* (Figure 3(c)). These results indicate that PNS regulates the intestinal microflora of DSS-induced colitis mice, forming a microbial composition that tends to be similar to that of normally fed mice. PCoA is the analysis of species diversity between samples. The closer the distance, the closer the species composition of the samples. The results show the changes in the overall structure of the mouse intestinal microflora after DSS induction (Figure 3(e)). We observed a clear clustering of the microbial composition of the normal, SASP, and H treatment groups. The hierarchical clustering tree (Figure 3(d)) reveals significant differences between the six groups and basically separates the samples processed by the H and M groups from the DSS group. This reflects that PNS treatment can adjust the DSS-induced colitis. Changes in the microflora of the intestinal microflora of mice, and make them tend to be the biological barrier of normal mice.

### 3.4. PNS Regulates Intestinal Epithelial Barrier Function

**Figure 4:**
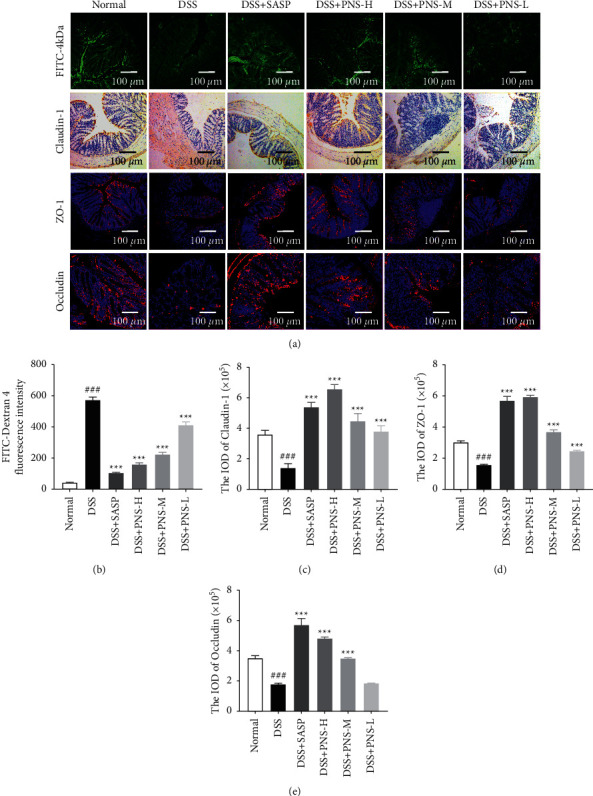
PNS improves intestinal epithelial barrier function. (a) Colon tissue section (FITC-4 kDa submucosal infiltration, Claudin-1 IHC staining, and representative photos of ZO-1 and occludin-1 IF staining), secondary antibody was used and observed by fluorescence microscopy (red staining). Nuclei were stained with 4,6‐diamidino-2-phenylindole (DAPI; blue staining); (b) the fluorescence intensity of FITC-D in serum at 4 h after administration (*n* = 9); (c) statistical histogram of Claudin-1 positive expression; (d) statistical histogram of positive expression of ZO-1; (e) statistical histogram of positive expression of occludin-1; the histogram data is calculated by selecting 10 random fields of view for each mouse, *n* = 3 mice for each group. ^#^*p* < 0.05, ^##^*p* < 0.01, and ^###^*p* < 0.001, versus the normal group, ^*∗*^*p* < 0.05, ^*∗∗*^*p* < 0.01, and ^*∗∗∗*^*p* < 0.001 versus the DSS group.

As an important line of defense for the human body, the intestinal epithelial barrier has an important role in preventing high-load bacteria and toxins from passing through the intestinal mucosa into the human blood circulation. FITC-Dextran 4 (4 kDa) was used to detect changes in epithelial permeability. There was a significant difference between the concentration of FITC-D in the serum of the DSS group and the PNS group (Figures 4(a) and 4(b)). The administration of SASP and PNS can significantly reduce the concentration of FITC-D in serum, which means that PNS reduces intestinal permeability and enhances the function of the intestinal mucosal barrier. As an important protein of the tight junction structure, the expression of claudin-1, ZO-1, and occludin was detected by immunohistochemistry and immunofluorescence methods, respectively. The expression of ZO-1, claudin-1, and occludin in the SASP group and the PNS group was significantly higher than those in the DSS group (Figures 4(c)–4(e)).

### 3.5. PNS Improves GC Depletion and Mucus Barrier Destruction Caused by DSS

**Figure 5:**
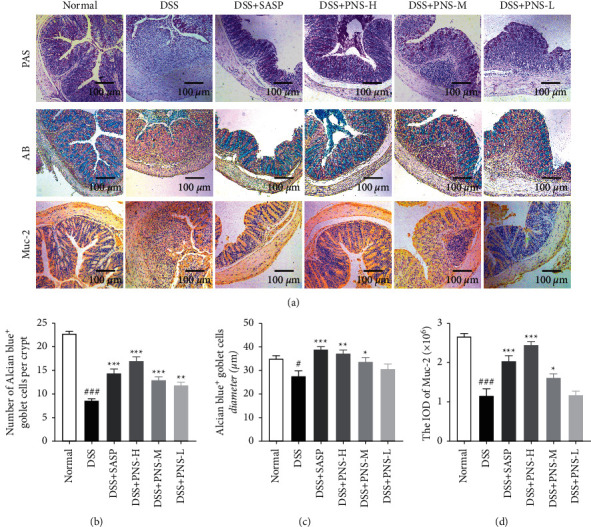
PNS improves GC consumption and mucus barrier destruction caused by DSS. (a) Colon tissue section (representative photos of AB staining, PAS staining, and Muc-2 IHC staining); (b) statistical histogram of the number of goblet cells; (c) statistical histogram of the size of goblet cells; (d) statistical histogram of Muc-2 positive expression. The histogram data is calculated by selecting 10 random fields of view for each mouse, *n* = 3 mice for each group. ^#^*p* < 0.05, ^##^*p* < 0.01, and ^###^*p* < 0.001, versus the normal group, ^*∗*^*p* < 0.05, ^*∗∗*^*p* < 0.01 and ^*∗∗∗*^*p* < 0.001 versus the DSS group.

The mucus barrier (mucus layer) is located between the microbial barrier and the intestinal epithelial barrier, which could resist endogenous or exogenous stimuli and microbial invasion in the intestine. And a complete mucus layer helps maintain the balance of the intestinal microbiota. The results showed that the number of GCs in the DSS group was significantly reduced (Figures 5(a) and 5(b)), some areas were significantly depleted, the residual GC became smaller (Figure 5(c)), and the crypt structure was destroyed. On the contrary, the number of GCs in the PNS group increased, and the arrangement was more orderly, suggesting that PNS has the effect of restoring the mucosal barrier function of the mouse intestine, making the number and size of goblet cells close to normal, and the crypt structure is restored. In the mice treated with SASP and PNS, the positive expression of Muc-2 was significantly increased (Figure 5(d)).

### 3.6. PNS Inhibits the Activation of Notch Signaling

**Figure 6:**
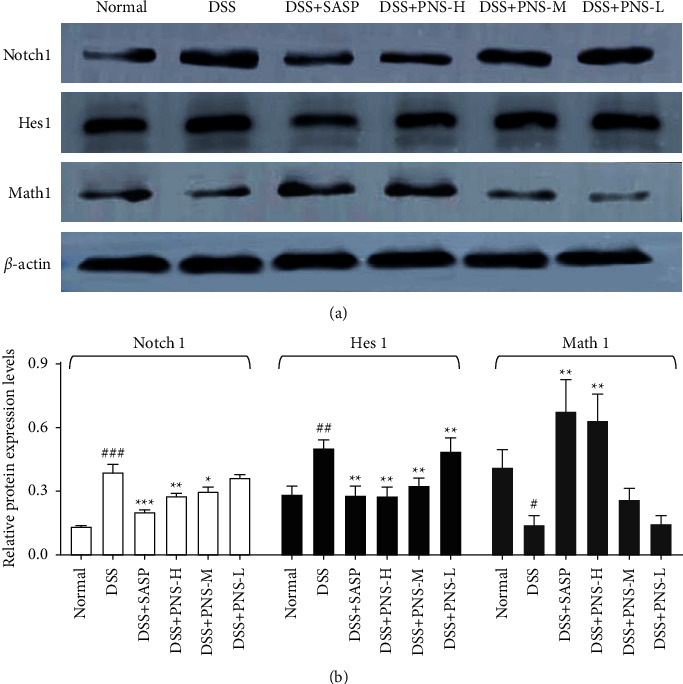
PNS can inhibit Notch signaling. (a) Representative western blot images for Notch1, Hes1, and Math1. (b) The protein expressions of Notch1, Hes1, and Math1. The histogram is the result of 3 independent experimental data. ^#^*p* < 0.05, ^##^*p* < 0.01, and ^###^*p* < 0.001, versus the normal group, ^*∗*^*p* < 0.05, ^*∗∗*^*p* < 0.01, and ^*∗∗∗*^*p* < 0.001 versus the DSS group.

To determine the mechanism by which PNS slows down DSS-induced colitis, we detected the Notch pathway, which reflects the most famous signaling pathway in the secretory lineage (including goblet cell differentiation) [20]. DSS treatment significantly increased the protein levels of the Notch pathway target genes Hes1 and Notch1 (Figure 6), indicating a strong activation of Notch signaling; in addition, the protein expression levels of Notch1 and its target gene Hes1 decreased significantly under the intervention of PNS. Consistent with expectations, the protein level of Math1 is inhibited by Hes1 and plays a key role in the differentiation of goblet cells. This indicates that the strong activation of Notch signaling induced by DSS was restored by the administration of PNS treatment.

## 4. Discussion

PNS is a traditional Chinese prescription. It has been widely used for acute or chronic purulent diseases in China. Recently, we proved that PNS has a significant antitumor effect on AOM/DSS-induced colon cancer and regulates the levels of inflammatory factors in the colon [21]. The four main absorption peaks of PNS determined by UPLC were identified with standard substances (Figure 1), including paeoniflorin, naringin, hesperidin, and neohesperidin. These compounds have been proved with different activities associated with the improvement of UC. According to the content of four ingredients in PNS, the dosage of corresponding compounds in the experimental mice was as follows: paeoniflorin (0.42∼1.68 mg/kg); naringin (1.05∼4.19 mg/kg); hesperidin (0.18∼0.70 mg/kg); and neohesperidin (1.09∼4.35 mg/kg). It has been reported that paeoniflorin (1 mg/kg) could improve inflammation in rats and reduce the expression of cytoinflammatory factors [22], and naringin (0.4∼40 *μ*g/kg) could prevent inflammation in mice exposed to endotoxin interference [23].

Paeoniflorin has a good anti-inflammatory effect on epithelial cells stimulated by LPS and significantly inhibits endothelial damage [24]. Naringin could reduce intestinal inflammation and increase the expression of TJs protein [25, 26]. Hesperidin and neohesperidin could maintain the diversity of colonic flora, maintain intestinal microecological balance, and play a beneficial role in intestinal barrier function and gastrointestinal inflammation [27–29]. Therefore, PNS may exert a beneficial effect on the DSS-induced colitis through multiple ways. DSS-induced colitis can directly damage intestinal epithelial-related cells, change the expression of TJs, cause erosion, and aggravate the loss of goblet cells and mucin [30], leading to typical mucosal barrier damage. The results of our study showed that treatment with PNS could significantly recover the weight (Figure 2(a)), decrease the DAI score (Figure 2(b)), and decrease the shortening of the colon (Figures 2(c) and 2(d)) in the DSS-induced colitis mouse model. The pathological tissue sections also showed that the PNS group could improve the morphology of epithelial cells (Figure 2(e)) and goblet cells (Figure 5(a)), and enhance the intestinal epithelial barrier function (Figure 4(a)).

The health of the intestine was closely related to the mucosal barrier. According to research, the onset of colitis is often accompanied by abnormal intestinal flora, leading to changes in the biological barrier [7, 31]. Imbalanced intestinal flora caused abnormal expression of TJs [32] and injured intestinal epithelial cells and goblet cells [30], and further reduced the Muc-2 secretion and the mucus barrier [33, 34]. With the damage of barrier function, the permeability of the intestine was increased, and the erosion of intestinal bacteria was promoted [35], which intensified the development of intestinal inflammation. At the same time, severe intestinal inflammation further exacerbates the changes in intestinal flora. Therefore, restoring the intestinal mucosal barrier in multiple ways is of great significance for the treatment of colitis. More and more studies believe that the imbalance of intestinal flora is the driving factor or trigger point of the onset of UC [36, 37]. In the composition of the intestinal flora of patients with colitis, the abundance of Bacteroidetes was decreased, while the abundances of Actinobacteria and Proteobacteria were increased, which was consistent with our results (Figure 3(b)). The changes in the flora were also closely related to the intestinal epithelial barrier and mucus barrier. For example, *Lactobacillus* increased the expression of Muc-2, improved the resistance of the intestine to pathogens, and induced the renewal and differentiation of epithelial cells [38, 39]. Odoribacter's fermentation product butyrate increased intestinal GC and promoted Muc-2 production [40]. *Escherichia-Shigella* and *Helicobacter* could invade the inner layer of mucus to reach epithelial cells. *Escherichia-Shigella* is involved in destroying the tight junctions of the intestinal barrier, increasing intestinal permeability and bleeding [41]. PNS regulates the balance of intestinal flora and is an important starting point for repairing the mucosal barrier. Its main ingredients include paeoniflorin, naringin, hesperidin, and neohesperidin which also play an important role in the regulation of intestinal flora [42–45]. This study confirmed that compared with the DSS group, the diversity of intestinal flora increased after PNS administration (Figure 3(a)), and the abundance of Bacteroides, *Staphylococcus*, *Odoribacter*, *Lactobacillus*, Isobacterium, and other bacteria increased, while *Escherichia-Shigella* bacteria, *Helicobacter*, *Streptococcus*, and other bacteria were significantly reduced (Figure 3(c)). These may be an important factor that PNS enhances the intestinal mucosal barrier.

Although we only discussed these four components, PNS also contains some polysaccharides and polyphenol components [46–50]. These substances also have anti-inflammatory, antioxidant, and regulating intestinal flora [50–56]. Maybe it is also the ingredient that PNS exerts its medicinal effect. The activation of the Notch signal determines the fate of intestinal epithelial absorption cells, and its inhibition affects the differentiation of secretory cells including goblet cells [20]. However, when intestinal epithelial cells are damaged, a large number of proliferating epithelial cells are needed to repair and rebuild the damaged part, and the Notch signal needs to be activated. The activation of the Notch signal inhibits the differentiation of GC and reduces the number of GC. In this case, the goblet cells will gradually decrease or even be depleted without being replenished and quickly consumed, resulting in reduced mucus secretion and destruction and disappearance of the mucus barrier [57], leading to microbial invasion and inflammation. Therefore, balancing Notch signals plays an important role in restoring the intestinal barrier function. The results of this experiment showed that PNS inhibited the expression of the Notch signaling pathway, reduced the expression levels of Notch1 and Hes1, and activated the expression of Math1. This could prevent the depletion of goblet cells and the low expression of Muc-2, which was consistent with the results of the previous AB staining. The restoration of the mucus barrier could protect the intestinal epithelial barrier and prevent harmful substances from being destroyed. These results indicated that PNS could inhibit the Notch signal, enhance mucus barrier, and promote the recovery of mucosa.

## 5. Conclusions

In summary, our current research results show that PNS can inhibit the Notch signaling pathway and play a role in multiple pathways to recover the intestinal mucosal barrier and improve colitis.

## Data Availability

The data used and/or analyzed during the current study are available from the corresponding author upon reasonable request.
